# Reducing Exposure to X-Ray in Patients With Conservatively Managed Distal Radius Fractures: A Closed-Loop Pilot Audit

**DOI:** 10.7759/cureus.31929

**Published:** 2022-11-27

**Authors:** Muattaz E Kazzam, Robert Clowes, Chris Wilson, Lucy Walker

**Affiliations:** 1 Trauma and Orthopaedics, Dorset County Hospital, Dorchester, GBR

**Keywords:** radiation dose reduction, cost reduction, x-ray evaluation, conservative management, hand-wrist radiograph, distal radius fractures

## Abstract

Introduction

British Orthopaedic Association Standards for Trauma (BOAST) guidelines state that a radiograph of the wrist at the time of removal of immobilisation is not required in conservatively managed distal radius fracture (DRF) patients unless there is clinical cause for concern. The aim of this pilot audit was to investigate local compliance with these guidelines.

Materials and methods

The first cycle of a retrospective audit was performed on conservatively managed DRF patients presenting between August and October 2021. An intervention was introduced in the form of education to highlight current guidelines. A second cycle was then performed prospectively on patients presenting between February and April 2022. Data was analysed to assess whether radiographs were taken at the time of cast removal, if the indication for the radiograph was documented and whether it affected the management plan.

Results

In the first cycle, 20 of 46 patients (43.5%) had repeat radiographs at the time of cast removal compared to 12 of 41 patients (29.3%) in the second cycle (p=0.170). None of the first-cycle patients had any documentation on the indication for radiograph at the time of cast removal and none of the radiographs altered the management plan. In the second cycle documentation on the indication for the radiograph was present for seven of the 12 radiographs and two altered the management plan.

Conclusion

Through education on adherence to national guidelines, the number of radiographs in patients with conservatively managed DRFs was reduced.

## Introduction

Distal radius fractures (DRFs) are one of the most common orthopaedic injuries in the world, with a rising incidence in part due to an increasingly ageing population [1-4]. Their incidence follows a bi-modal distribution, commonly associated with high-energy trauma in younger patients and lower-energy trauma in elderly patients [2].

The management of DRFs is varied. Non-operative treatment options include closed reduction and immobilisation in plaster or a splint. Operative treatment options most commonly include open reduction and internal fixation, percutaneous fixation or external fixation [5]. Decision-making between conservative and operative treatment options will depend on patient and fracture characteristics. In addition, rates of operative treatment vary geographically [4,6].

In the United Kingdom, the British Orthopaedic Association Standards for Trauma (BOAST) has set out guidelines for the management of DRFs, including guidance on follow-up radiographs [7]. These state that no radiograph is required at the time of removal of immobilisation unless there is any clinical concern. Although evidence for this in the literature remains limited, some studies have been carried out to support that these radiographs are unlikely to alter treatment strategy; a study by Weil et al. [8] found that of 804 conservatively managed DRFs, 464 received follow-up radiographs of which only 12 (2.7%) altered the management plan. Nine of those (2.2%) were categorised as routine. Another study by Eastley et al. [9] looked at 138 conservatively managed, uncomplicated and extra-articular DRFs. They found no difference in grip strength or range of movement (ROM) between patients who had a radiograph later than two weeks post-injury and those who did not. None of the patients who had radiographs later than two weeks post-injury required surgical intervention.

The aim of this pilot audit was to assess compliance with BOAST guidelines with a view to reducing the number of radiographs at cast removal that are not clinically indicated. This may lead to a reduction in healthcare costs and the workload of the radiology department, as well as reduce X-ray exposure to patients.

## Materials and methods

Ethical approval

The audit was approved and registered with the local clinical audit department (audit number: 5389). As the analysis in this study was a retrospective review of already available, anonymised data as part of clinical auditing, research ethics committee approval was deemed not to be required.

Audit design

A closed-loop audit was carried out in the Trauma & Orthopaedic department of a district general hospital in the United Kingdom. This study identified patients with DRFs from referrals to the virtual fracture clinic (VFC). The VFC is an orthopaedic consultant-led clinic which reviews referrals from the emergency department and minor injury units for consideration of further orthopaedic management and follow-up. Referring clinicians follow set guidelines to identify cases appropriate for referral; in the case of DRFs, this includes closed, neurovascularly intact injuries with radiographic parameters appropriate for non-operative management.

The first audit cycle identified patients with DRFs referred to the VFC between 1st August to 30th October 2021. Following data collection and analysis, an intervention was then introduced in the form of education to the local orthopaedic department. This included a presentation of the results and highlighting the BOAST guidance on follow-up radiographs of DRFs at the departmental morbidity and mortality meeting. A second audit cycle was then carried out on DRFs referred to the VFC between 1st February to 30th April 2022 to assess for any change in compliance with guidelines.

Data collection

Electronic documentation of patients in our pre-existing virtual fracture clinic (VFC) referral database was reviewed to identify all those with DRFs. This was done retrospectively in the first cycle and prospectively in the second cycle. Patients were excluded if they were under the age of 18 years or had open physes, underwent operative treatment as primary management, had splint rather than cast immobilisation, were lost to follow-up, or if they were out of area.

Primary outcome measures included whether a radiograph was taken at the time of plaster removal, if the indication for the radiograph was documented, and whether it affected the management plan. Secondary outcome measures included:

• Was manipulation performed in the emergency department?

• Number of face-to-face clinic reviews

• Total number of post-injury radiographs

• Length of time in a cast

• Any documented complications

• Fracture classification

Sources of data included a review of available radiographic imaging, emergency department notes, VFC referral letters and orthopaedic fracture clinic letters. The end-point of data collection for each patient was at the time of discharge from the fracture clinic. This included discharge to hand therapy as well as referrals to a hand surgeon for consideration of further management of complications. Fractures were classified according to the AO classification system for DRFs into types A, B and C (extra-articular, partial articular and complete articular fractures respectively) as well as their nine groups; subgroups were not included [10].

Data analysis

Outcome measures were compared between patients in the first and second audit cycles to assess the effect of the intervention on compliance with the BOAST guidelines. Additionally, outcome measures were compared within each cycle between patients who received radiographs at plaster removal and those who did not. Chi-squared tests were used for the analysis of categorical data. For outcome measures where sample sizes were too small for analysis with the Chi-squared test, Fisher's exact test was used. Two-tailed unpaired t-tests were used for continuous data. Statistical significance was set at p ≤ 0.05.

## Results

In the first audit cycle, the final cohort had 46 patients with DRFs including nine males and 37 females. Mean age at the time of injury was 64.1 years (range: 18-94). Of these, 20 patients (43.5%) had a repeat radiograph at the time of removal of plaster immobilisation. None of these had any documentation regarding the clinical indication of the radiographs and none of the radiographs altered the management plan.

In the second audit cycle, the final cohort had 41 patients with DRFs with five males and 36 females. Mean age at the time of injury was 70.6 years (range: 19-91). In comparison to the first cycle, in the second audit cycle 12 patients (29.3%) had a repeat radiograph at the time of removal of plaster immobilisation (X^2^ = 1.88, p = 0.170). Seven of these had documentation regarding the clinical indication of the radiographs and two of the radiographs altered the management plan.

Comparisons between the two cycles for all patients are summarised in Table 1. Comparisons between patients who did and did not receive radiographs at plaster removal in the first and second audit cycles are summarised in Table 2 and Table 3 respectively. The distribution of fracture types according to AO classification in both audit cycles is summarised in Table 4.

**Table 1:** Comparison of outcome measures between patients in the first audit cycle and the second audit cycle. *: p-value calculated using Fisher’s exact test

	First audit cycle (N = 46)	Second audit cycle (N = 41)	p-value
Age (mean)	64.1	70.6	0.075
Gender (Male:Female)	9:37	5:36	0.350
Patients with radiographs at the removal of immobilization (N (%))	20 (43.5%)	12 (29.3%)	0.170
Documented indication for radiograph at the removal of immobilization (Yes:No)	0:20	7:5	<0.001*
Manipulation under anesthesia (Yes:No)	15:31	19:22	0.190
Length of time in cast (mean in days)	36.3	38.5	0.133
Number of X-rays (median)	4	3	0.599
Number of clinic reviews (median)	2	3	0.667
Complication/ongoing review (N (%))	1 (2.2%)	3 (7.3%)	0.339*

**Table 2:** Comparison of patients who received radiographs at the time of removal of plaster immobilisation and those who did not in the first audit cycle. *: p-value calculated using Fisher’s exact test

	No radiograph with cast removal (N=26)	Radiograph with cast removal (N=20)	p-value
Age (mean)	67.3	59.8	0.164
Gender (Male:Female)	6:20	3:17	0.494
Manipulation under anesthesia (Yes:No)	7:19	8:12	0.348
Length of time in cast (mean in days)	36.5	36.1	0.828
Number of X-rays (median)	3	5	<0.001
Number of clinic reviews (median)	2	3	0.094
Complication/ongoing review (N (%))	0 (0%)	1 (5%)	0.435*

**Table 3:** Comparison of patients who received radiographs at the time of removal of plaster immobilisation and those who did not in the second audit cycle. *: p-value calculated using Fisher’s exact test

	No radiograph with cast removal (N=29)	Radiograph with cast removal (N=12)	p-value
Age (mean)	73.6	62.6	0.035
Gender (Male:Female)	2:27	3:9	0.107
Manipulation under anesthesia (Yes:No)	15:14	4:8	0.283
Length of time in cast (mean in days)	38.5	38.6	0.969
Number of X-rays (median)	3	5	0.013
Number of clinic reviews (median)	3	3	0.451
Complication/ongoing review (N (%))	1 (3.5%)	2 (16.7%)	0.200*

**Table 4:** Distribution of fracture types according to AO classification in both audit cycles

Fracture Classification	First Audit Cycle (N=46)			Second Audit Cycle (N=41)		
	No radiograph with cast removal (N=26)	Radiograph with cast removal (N=20)	Total	No radiograph with cast removal (N=29)	Radiograph with cast removal (N=12)	Total
A1 - radial styloid avulsion	0	0	0	0	0	0
A2 - simple	11	4	15	12	2	14
A3 - wedge/multifragmentary	5	7	12	10	2	12
B1 - sagittal	5	4	9	3	3	6
B2 - dorsal rim	1	1	2	0	1	1
B3 - volar rim	0	0	0	0	0	0
C1 - simple articular and metaphyseal	2	1	3	1	2	3
C2 - metaphyseal multifragmentary	2	3	5	3	2	5
C3 - articular multifragmentary, simple or multifragmentary metaphyseal	0	0	0	0	0	0

Of the 46 patients in the first audit cycle, 45 were discharged with no documented complications. One patient (52-year-old male, fracture type B2) remained under ongoing review due to ongoing ulnar-sided wrist pain as well as concerns of scapholunate ligament/triangular fibrocartilage complex injuries for which they were awaiting a diagnostic arthroscopy. This patient did have a radiograph at the time of removal of immobilisation which showed some worsening dorsal tilt but did not change the management strategy of the DRF.

Of the 41 patients in the second audit cycle, 38 were discharged with no documented complications. Three patients were under ongoing review:

• 62-year-old female (fracture type A2) who had mild carpal tunnel compression symptoms directly following injury and ulnar-sided pain. She had an X-ray at the time of removal of the cast which showed the distal radius had healed in a dorsally angulated position. She was discharged to hand therapy at eight weeks post-injury with a view for a possible future osteotomy and/or carpal tunnel release if symptoms were ongoing.

• 63-year-old female (fracture type B2) seen in clinic at one-week post-injury and again at 19 days post-injury for fracture displacement monitoring. Radiographs at 19 days showed significant shortening and dorsal angulation with an intra-articular split. A decision was made to continue with conservative management. Plaster immobilisation was removed at 51 days post-injury and a radiograph at removal, taken due to localised tenderness, showed radial malunion and ulnar impaction. The reason for prolonged cast immobilisation was unclear. She had limited improvement with hand therapy and was referred to a hand surgeon for consideration of osteotomy/triangular fibrocartilage complex (TFCC) management.

• 69-year-old female (fracture type A3) discharged at six weeks with no pain or tenderness, therefore no radiograph was taken at the removal of plaster immobilisation. Represented three days later with pain and swelling of the affected wrist, without a clear history of trauma but significant cognitive impairment was noted. A repeat X-ray showed that the fracture had not yet united. The patient was followed up two weeks following this with a repeat radiograph showing signs of fracture healing; therefore, plaster immobilisation was taken off and she was discharged to hand therapy.

## Discussion

The main finding of our study was that through education, adherence to national guidelines on follow-up radiographs in DRFs can be improved, resulting in a reduced number of routine radiographs at the removal of plaster immobilisation. Our findings also suggest that in the absence of clinical concerns, routine radiographs at the removal of immobilisation seldom alter the management plan.

Current BOAST guidelines currently advise that no radiograph is required at the time of removal of immobilisation of DRFs unless there is a clinical concern [7]. Of the 32 radiographs taken at the time of removal of plaster immobilisation in both cycles, only three changed the management plan of the DRF. In two of these cases, radiographs were taken due to documented clinical concerns, hence the guidelines were adhered to, and complications were correctly identified. In the one case that did not have a radiograph at plaster immobilisation removal and returned with a complication, the history of cognitive impairment makes interpreting this case more difficult. On the one hand, there were no clinical concerns on the removal of the plaster and as such not taking a radiograph was in adherence with the guidelines. Furthermore, in the context of cognitive impairment, a possible missed traumatic insult to the fracture following plaster removal can be difficult to rule out.

Taking a radiograph may not alter the management plan at the time; however, retrospectively it may possibly provide a benefit from a medicolegal aspect. In the National Health Service (NHS) in the United Kingdom, the frequency of successful litigation compared to the number of orthopaedic procedures performed remains low but highly costly; between 2000 and 2006, more than 321,695,072 US dollars was paid out in orthopaedic surgery-related settlements [11]. In DRFs, malunion represents one of the most common reasons for litigation [11-13]. In this context, avoiding litigation should be considered as a possible reason for clinicians requesting routine radiographs after removal of immobilisation as they may feel it further supports their decision to continue with non-operative treatment. Additionally, in the case of further complications, it may be a form of baseline imaging prior to referral/discharge to hand therapy. The impact of this medicolegal aspect on the clinician’s decision to request routine radiographs should be considered and could be further investigated in future studies in the form of anonymised clinician surveys.

Reducing routine follow-up radiographs is likely to confer a cost benefit to the health service provider. In our audit, education on national guidelines reduced routine radiographs at the removal of plaster from 44.7% to 29.3%. The cost of a wrist radiograph in the NHS between 2020/21 was reported to be £59 [14] and with a high incidence of DRFs in the United Kingdom [15,16], reducing routine radiographs is likely to provide a significant cost-saving to the health service. However, further analysis in the form of a cost-benefit analysis is required to determine the true extent of this.

The distribution of different fracture patterns was similar in both audit cycles overall. In the second audit cycle, fewer patients with extra-articular DRFs (A2 and A3 types) had radiographs at the time of removal of plaster compared to the first audit cycle. A potential reason for this may be that following increased awareness of guidelines, clinicians felt more comfortable not requesting radiographs at cast removal for more simple fracture patterns in the absence of clinical concerns. It should be noted that in our study, two out of four patients with complications had A2 and A3 type DRFs and thus, this likely reflects the need to assess the clinical context rather than relying on radiographic fracture classification to decide when radiographs at plaster removal are indicated.

There were some limitations to our study. Due to the timeframe of the audit, the sample size in our study was small and the results should be interpreted with caution. Using VFC referrals to identify DRFs incurs the possibility of selection bias. Unstable and/or intra-articular fracture patterns are mostly referred directly to the on-call orthopaedic team and discussed in the trauma meeting where a decision of operative vs. conservative management is made; conservatively managed cases of DRFs from this pathway were not included in our study. However, our local emergency department follows strict criteria on which cases are suitable for referral to the VFC and include cases unlikely to require operative intervention.

Finally, due to time constraints and the retrospective nature of the study, we did not collect data on long-term outcomes and complications following the removal of immobilisation for all our patients. Although we found most of the radiographs at that stage did not alter the management strategy, the effect of reducing follow-up radiographs on long-term complication rates remains unclear. However, there is evidence in the literature to indicate a low rate of long-term complications in appropriately managed DRFs, with a change in management strategy unlikely to be required. A recent study in working-aged patients by Hevonkorpi et al. [17] found that in 179 conservatively managed DRFs, only two patients required operative intervention at a stage later than three weeks post-injury. Furthermore, multiple studies suggest a low rate of operative intervention for DRFs initially managed non-operatively in elderly patients [18-21].

## Conclusions

The findings of our study suggest that through education on the national guidelines for DRFs, we were able to reduce the number of routine radiographs at the time of removal of plaster immobilisation of the conservatively managed fractures. This is likely to have translated into a reduction of financial and workload burden on outpatient orthopaedic services. The authors suggest a similar audit strategy for clinicians to assess and/or improve adherence to guidelines in their local orthopaedic units.
